# Notes and News

**Published:** 1892-10-15

**Authors:** 


### Notes and News.

Collected and Communicated.

We are glad to learn that the idea of a Life-Boat Saturday is gaining ground in Scotland. A trades demonstration was held in Dundee, in aid of the funds of the institution, and a suggestion to organize something of the kind has been made in Glasgow.

The *Leisure Hour* for this month contains an interesting article on "Pond-Hunting for the Microscope." We are given practical and simple directions for the management of a micro-aquarium. The writer is enthusiastic himself, and deals with his subject in a manner to inspire interest in those already slightly smitten with the fascinations of that revealer of wonders, the microscope.

Princess Louise opened the new surgical and pathological pavilions, which form part of the Jubilee extension scheme of the Aberdeen Royal Infirmary, on the 4th inst. By this addition the number of beds available will be increased from 175 to 240. The most elaborate and tasteful decoration in the streets and in the infirmary was arranged, and a large party was entertained at luncheon. Beautiful bouquets were presented to Princess Louise and to Princess Henry of Battenberg, who accompanied her.

The most unsatisfactory of all the "snowball" cases yet revealed—that set rolling by two women in domestic service—has now been inquired into; for some time, the results of the inquiry failed to bring any real proof to light of how the money had been utilised. The Young Women's Christian Association, in whose behalf the snowball was commenced, knew nothing of the affair from first to last. The only person who did seem to know appears to be a Mr. Caldwell, giving an address at a "missionary bureau" at 186, Aldersgate Street. Here all the money, it is stated, was sent by the two young women. Persistent inquiry has drawn from Mr. Caldwell a statement of accounts. £317 is acknowledged as having been received, and has provided seven missionaries with funds. The number of letters received is most remarkable. Over three hundredweight of envelopes alone, containing these letters, have been sold.

The Chairman and Treasurer of the Metropolitan Hospital, Kingsland Road, make a special appeal to the public on behalf of their institution. They state that whilst they are able to accommodate 160 patients, it has been only possible to admit 78 at one time. It will be necessary to close two out of the five wards unless a sum of £5,000 is forthcoming by the end of the year.

Now that the North-Eastern Hospital is really completed at Tottenham, the temporary status of the structure is being especially drawn attention to by the Chairman of the Middlesex County Council. He evidently suspects the thin end of the wedge is well in, and loses no time in reiterating before quite too late the many objections to the Tottenham site. He predicts popular prejudice as quite insurmountable, whilst admitting that no evil effects from the locality of the fever hospital at Islington, a far more crowded neighbourhood, have been shown. Who would be the promoter of a fever hospital? A more unpopular and onerous task is hardly to be conceived.

The opening of the session at the Meath Hospital was marked by Mr. Hepenstal Ormsby's inaugural address, in which he dealt with the grievances of the medical profession. The much-enduring Irish Dispensary doctors came first on the list. A special plea for a superannuation allowance in these cases was made. The hard fate of dispensary doctors, as they existed but a short time back, was long unrealised; now public feeling is awakening to a sense of the real grievances of this part of the medical profession. Mr. Ormsby spoke with some force on the injustice of the want of recognition afforded to the Irish diploma, with but two exceptions, in England. He also considered that the Army Medical Service had every claim to be regarded as and entitled a Royal corps, and further suggested that adequate consideration to the rights of the profession could best be attained by Parliamentary representation.

The Secretary of the Mansion House Fund for the relief of the inhabitants of St. John's, Newfoundland, has now received an account of the expenditure of the money sent from England. Up to the present time £36,941 has been received in money, and £14,782 in goods. Large as the sum appears the wants that have to be provided for are still greater, and the coming winter will tax all resources to the utmost. £19,220 has been already expended, and £17,720 remains in hand. At first gratuitious relief was afforded, but gradually this was conferred on the aged and infirm alone, as the able-bodied found employment. Many were provided with materials wherewith to erect temporary dwellings. Fifty thousand dollars has been especially reserved for distribution amongst single ladies, clerks, and others, who has fallen from circumstances of comfort to indigence, this being especially recommended by the Mansion House Committee. For such persons, also, it is proposed to erect houses, which will be let to them at low rents. It will be seen that so large a scheme of relief as the St. John's Relief Committee have before them will require a very considerable expenditure, and the London Committee doubtless have still further call for the funds at their disposal.

